# Mind and material engagement

**DOI:** 10.1007/s11097-018-9606-7

**Published:** 2018-12-01

**Authors:** Lambros Malafouris

**Affiliations:** Keble College & Institute of Archaeology, University of Oxford, Oxford, UK

**Keywords:** Material engagement theory, Things, Metaplasticity, Pottery making, Cognitive archaeology, Enactivism

## Abstract

Material Engagement Theory (MET), which forms the focus of this special issue, is a relatively new development within cognitive archaeology and anthropology, but one that has important implications for many adjacent fields of research in phenomenology and the cognitive sciences. In *How Things Shape the Mind* (2013) I offered a detail exposition of the major working hypotheses and the vision of mind that it embodies. Here, introducing this special issue, more than just presenting a broad overview of MET, I seek to enrich and extend that vision and discuss its application to the study of mind and matter. I begin by laying out the philosophical roots, theoretical context and intellectual kinship of MET. Then I offer a basic outline of this theoretical framework focusing on the notions of *thinging* and metaplasticity. In the last part I am using the example of pottery making to illustrate how MET can be used to inform empirical research and how it might complement new research in phenomenology and embodied cognitive science.

## Introduction: walk the line

Let’s begin with a simple task. Take pen and paper. Draw a line. The sketch of any form will do. Just leave a trace. Make a mark. What constitutes an adequate description of, and how do we account for the process by which skills, hands, instruments and materials intersect to create a trail of ink on the paper’s surface? Our most habitual actions (psychical or physical) are experienced and become constituted where brain, body and culture conflate. Yet, finding adequate ways to describe this conflation, even in the simple case of line-making, pose a great challenge.^1^ Where do we start delineating the boundaries of the marks our moving hand leave on a surface? What kind of mental processes and forms of representation can account for the origins and endings of the simple line we have drawn? Consider three common ways to describe the line. The first way is to think of it as an action: the drawing of a line. The second way is to think of it an object: a line drawn on paper. The third way is to think of the line as a sign: the index of our moving hand or perhaps the trace of a creative gesture.^2^ Ontologically speaking, those three ways of seeing the line are inseparable. Each one of those ways supports, informs, constraints, causes and complements the other. To grasp their unity is to attend the cognitive life of the line. Attentiveness to the cognitive life of the line will allow us to see sentience in the trail of ink. Yet, more often than not, we seem to resist this realization. The preferred analytical convention is to break the line’s cognitive life into pieces: first by separating ourselves from the line and then by seeing the line as the ‘external’ product of a sentient ‘internal’ process. As ‘modern’ human observers we have learned to see the line where the movement stops and the drawing ends. We have also develop the conviction that some pre-formulated ‘idea’ or ‘mental representation’ of a line inside our head precedes and causes the materialization of the line in the outside world. The anthropologist Tim Ingold refers to this representational tendency as inversion. The logic of inversion, characteristic of modernity, manifest as an attempt to reconfigure the relational matrix of the world we live into a series of internal representational schemata of which our actions are but an outward expression: “Through inversion, beings originally open to the world are closed in upon themselves, sealed by an outer boundary or shell that protects their inner constitution from the traffic of interactions with their surroundings” (Ingold 2010, 355). Most of our troubles with the nature and evolution of human cognition depend and stem from this representational logic of inversion that sets up the artificial opposition between mind and matter.

## What if the mind has no a priori location?

Setting the boundaries of the human mind was never easy. Specifying the conditions under which a process falls on the ‘inside’ or on the ‘outside’ of those boundaries even more so. As I said, the conventional way of dealing with this problem, marking the mental and delineating those boundaries, has been to divide the world a priori in two parts, a mental part and a physical part. The mental part is the sentient part that thinks by re-presenting the other physical part that is lacking this precious ability. In one sense, the mental part deals with what is absent (representing, remembering, imagining) and the physical part with what is present (in the ways we touch the world and the world is touching us). For instance, the line in our example belongs to the physical part as the end-product of a human intention that originates in the mental part. Perhaps this bifurcation of the world works well within the metaphysical confines of a representational space where lines and material forms have no real life and where the physicality of traces does not matter. Yet, this separatist logic fails in most real-life situations where our ways of thinking, of making, and of doing, are inseparably linked as part of an evolving material ecology.

Cognitive archaeology (which is the field that examines the macro-history of human thinking: how it becomes constituted, transformed and reproduced in different contexts and configurations of brain-body-material environment in the course of human becoming) offers plenty of evidence to support this basic claim against the separation of thinking inside the head and acting inside the world.^3^ Perhaps this claim is less obvious for other disciplines that do not afford a deep time perspective and lack any particular expertise or familiarity with the causal efficacy of material culture in human cognitive life. I should make it clear, then, that not just the size of our brains and the shape of our bodies but our ways of thinking and of socializing are rooted in those elementary gestures of enactive material signification. Line making is just one elementary example of that process. Humans become ‘*through a saturated, situated engagement of thinking and feeling with things and form-generating materials*’ (Malafouris 2014, 144 italics in the original). I call that process creative *thinging* and will return to exemplify its meaning below. Suffice it for now to say that from the earliest lithic ecologies to the latest digital ontologies this process is at the heart of human evolution. Humans think by constructing signs, by drawing lines and by leaving memory traces. They do all that primarily by means of their moving bodies, especially their hands. This is not to say that the signs we make or the lines we draw merely ‘represent’ or ‘reflect’ intelligence. The ‘reflected’ intelligence is not hidden away in some separate ‘mental’ realm inside the skull. The moving hand and its material traces do not just externalise the internal workings of a mind. Instead, intelligence is enacted through them; it proceeds along lines and material signs of one kind or another. For instance, the making of a stone tool is not the product of thinking; it is a way of thinking. When we look at a stone tool we don’t simply see the externalization of form, skill or memory; rather we observe how the affordances of stone make possible for human bodies to learn and to remember skills, to sense causality, or to enact intentions. In short, within a lithic ecology, stone tools bring forth and constrain the organism’s possibilities for action and imagination. In that sense the process of thinking is effectively turned inside out. Our forms of bodily extension and material engagement are not simply external markers of a distinctive human mental architecture. Rather, they actively and meaningfully participate in the process we call mind.

This basic idea of a mind not limited by the skin has a long ancestry in various intellectual traditions. We are mistaken, the early pragmatist and semiotician Charles Sanders Peirce reminds us, “to conceive of the psychical and the physical aspects of matter as two aspects absolutely distinct. Viewing a thing from the outside, considering its relation of action and reaction with other things, it appears as matter. Viewing it from the inside, looking at its immediate character as feeling, it appears as consciousness.” (Peirce 1994, 6.268). Indeed, from John Dewey’s ‘transactional’ sense of ‘situation’, to Alfred North Whitehead’s process philosophy of becoming, to Henri Louis Bergson’s idea of ‘creative evolution’ (1998)[1911]), to the phenomenology of Merleau-Ponty (1962, 1968), to the more recent work in ecological psychology of Gibson (1977, 1979) and Gregory Bateson’s (1973; 1979),^4^ critiques of the oppressive modernist alienation of the mind from the material world have been gathering momentum throughout the twentieth century. Today, the debate continues more intensely than ever, with new theoretical and empirical work on enactive, distributed, embodied and extended cognition (Varela et al. 1991; Hutchins 1995, 2010a; Clark 1997; Chemero 2009; Thompson 2007; Hutto and Myin 2013; Gallagher 2017; Laland 2017).

What then if, trying to answer the fundamental questions about the nature of human intelligence, we start from the assumption that the mind has no a priori location or place of origin? What if, adopting a point of view well supported in cognitive archaeology and anthropology, we assume that the stuff of mind do not exist only inside the head but can be found also, if not primarily, inside the world?

## Where brain, body and culture conflate

Let me rephrase those questions returning to the example we have started this article with: what if the lines we leave behind in drawing, like the paths we lay down in walking, are marking the mental? Imagine we were to re-describe the process of line-making by focusing on the moment where the pen stops but is still touching the paper’s surface. It seems that, for that single moment, all three aspects of form-making, i.e., the line as gesture, the line as object and the line as a trace co-exist. They are no longer seen as separate, instead, they can be seen as a transformative, constitutive intertwining of neural, bodily, and material recourses. What then if we try to create a theory of human intelligence taking this enactive co-habitation of marks and traces (both neural and extra-neural) as the point of initiation?

Accepting that human thought processes are better described as *hylonoetic*^5^ semiotic fields — a mindscape constituted by bodily practices and artefacts— places us in position to restate the problem of the interaction between cognition and material culture. Not just the lines we draw on paper, but also our imaginary lines, those that connect our past with our present and possible future and allow us to become the self-conscious beings we are, exist in the middle space where brain, body and culture conflate: never entirely mental, in the ‘internal’ sense, and never just material, in the ‘external’ sense. Neither mind, in the cognitivist sense, nor matter in the materialist sense. What kind of theory can describe that middle?

Material Engagement Theory suggests a way of looking at, and sets out a possible pathway to approach this middle in-between space where brain, body and culture conflate (Malafouris 2004, 2013). Grounded in the anthropological archaeology of the mind (Malafouris 2004; Malafouris and Renfrew 2008, 2010; Renfrew 2004; Renfrew et al. 2008; Iliopoulos and Malafouris 2014), the material engagement approach is committed to observing and describing cognitive life as we find it, enacted inside the world by the people of different places and times (past and present). Based on that commitment the material engagement approach comprises some radical ideas, perspectives and epistemic constrains, that allow us to take seriously the materiality of mind-stuff in the way we approach the study of human thought. Below I offer a summary of some major differentiating features.

### On boundaries and mind-stuff: a process archaeology of mind

One important characteristic of the Material Engagement approach that follows naturally from our previous discussion, lies in its conviction that in order to study the cognitive life of any species we need to understand the lines, forms and material traces left or made in the course of it’s becoming. That is, we need to follow the variety of mind-stuff as they fold and unfold, entangle and disentangle, in different temporal and spatial scales of a species’ phylogeny or ontogeny. With mind-stuff I refer to the dynamic ensembles, flows and configurations of matter and energy by which sentient creatures become organized and relate to their surrounding environment and to each other. We should not forget that, what we try to articulate when we use the term ‘mind’, can be better described as a verb. There is no such universal thing as ‘the mind’, rather there is a variety of human (or by extension non-human) *ways of thinking* enacted by specific bodies in specific situations (historical, social or cultural)*.* What we call mind is a ‘process’ constituted by the continuous recycling and re-organisation of mind-stuff, i.e., a cognitive becoming. Thinking, like form-making, exists in a state of perpetual movement. Minds never stop minding. Minds always become. This applies to every sentient organism but is especially true in the case of humans given the profound plasticity and immense variety of the material forms that we make (Idhe and Malafouris 2018; Ingold 2013).

MET proposes that we can only understand human beings (what is it to be human) by understanding the modes of *human cognitive becoming* (how human minds become) (Gosden and Malafouris 2015; Malafouris 2015, 2016a, b). These modes of becoming are actualised at different temporal and spatial scales (personal, peripersonal, and extrapersonal) by means of material engagement. That also means that MET, although sensitive to the faster timescales of neural events, is mainly concerned with slower timescales characteristic of human activity both at the developmental and the evolutionary scale (see also Aston this issue). The unhelpful antinomies of mind/matter, nature/culture and people/things now give way to a more productive focus on the ways materiality becomes entangled with our lived experience and thinking. We have a plastic mind, inextricably intertwined with the plasticity of culture. I call that special feature of human becoming metaplasticity (Malafouris 2010a, 2013, 2015). Material Engagement Theory takes this metaplastic recursive relationship between brains, bodies and things as the main analytical unit for the study of human thought processes. Contrary to methodological individualism, that is the view that anything mental must refer to, and is explained by processes internal to the individual, the study of metaplasticity demands an action-centered methodology especially adapted for handling the complexities of human cognitive becoming in a variety of socio-material settings and across the scales of time. As the anthropologist Edwin Hutchins points out “the proper unit of analysis for cognition should not be set a priori, but should be responsive to the nature of the phenomena under study” (Hutchins 2010b, 426). MET provide such a flexible unit of analysis that allows to view the mind as situated within and constituted by the material world rather than merely being *about* the world.

Perhaps, sometimes, for specific questions and phenomena under investigation, the right boundaries must be closed, narrow and specific. Cognitive neuroscience, to give one example, operates on that assumption focusing on the study of so-called ‘neural’ representations and their complex networks of activation and de-activation human brain. Methodologically speaking, this closure makes good sense if you’re just interested in the human brain and what flows therein, which now can be measured by means of blood oxygen level – dependent (BOLD) functional magnetic resonance imaging. But the narrow substitutional logic of such a reductionist approach embodies a neurocentric attitude that can mislead us to think that all that really matters to study the mind is to understand the nature, formation and processing of internal mental representations. This threatens to turn human cognitive life into a lifeless abstraction. On the contrary, human cognitive life extends beyond skin and skull. As a result, it is important, when we decide exactly how and where to set the boundaries of the cognitive phenomena we seek to investigate, not to “cut lines of interaction in ways that leave key aspects of the phenomena unexplained or unexplainable” (Hutchins 2010b, 426). One of the major challenges for MET is to study the changing nature of those boundaries in the course of human becoming and the role they may have played in determining the possible interactions across the world/body/brain system.

Obviously, my previous remark do not mean to question the neural bases of human thinking and feeling or the immense contribution that a contextualized critical neuroscience can make in delineating how the varieties of human thought and consciousness are supported and mediated by the brain. I do, nonetheless, seek to underline that from the vantage point of a ‘process archaeology of mind’ (Gosden and Malafouris 2015; Malafouris 2015, 2016a, b; Malafouris and Koukouti 2018) one can hardly find any convincing reason – besides convention – why the process we call mind should be restricted to neural or representational ‘events’ occurring inside an individual’s brain. Specifically for MET, as with many other enactive and distributed approaches to the study of mind, there can be no *a priory* separation between what is ‘out there’ and ‘in here’ with respect to the boundaries of skin and skull. The skin cannot act as a barrier between the mind and the material world. Minds are not confined within individual brains, bodies or any other isolated locale. Rather, we should see the ‘mental world’ as immanent in the relations and transformations that allow human beings to reach out and to engage their surrounding environments. Hutchins (2010a), inspired by Bateson (1973), uses the term *cognitive ecology* to describe this kind of relatedness. The challenge for us, then, becomes one of devising methods for tackling this “relational domain” as it becomes realised in different contexts of situated action.

Of course, the role of the brain as an assemblage of neural activation and de-activation patterns remains central. However, the centrality of the brain does not lie in its ability to constitute mentality by internalising and representing the world; rather, it lies in the ability of the brain to connect, to attend, to respond, to attune and relate to the world using its extraordinary plasticity and sensitivity. In other words, brain operations are inseparable from the rest of the body and its surrounding relevant environment. Importantly, there is no central executive. No single part of this dynamical system is responsible for central processing. What is often described or seen as central processing is in fact an attribution of agency. The patterns of neural activation that one may observe and associate with a specific pathway of world-engaging action do not ‘represent’ that pathway, rather, they simply correspond with it. This correspondence can take in time and, for specific situations, a variety of forms (affective, semiotic, social or aesthetic) but it can never emerge in the absence of some minimal material engagement. The basic process described here is very similar to what Gallagher and Allen refer to “ongoing predictive engagement (PE)—a dynamical adjustment in which the brain, *as part of and along with the larger organism*, actively responds in ways that allow for the right kind of ongoing attunement with the environment—an environment that is physical but also social and cultural”(2018, 2634, emphasis in the original). The material ecology and the cognitive ecology of our everyday worldly engagements are in fact inseparable. MET puts special emphasis in trying to understand specific instances of these bio-social relations, their material constitution and transformations in human becoming.^6^ This brings us to the issue of things and the notion of *thinging.*

### Thinking as thing-ing

I said before that mind-stuff do not have fixed locations or set properties: they equally pertain to brains, bodies and things. A neural activation pattern, a movement of the hand, a line produced on a piece of white paper: they are all mind-stuff. This, of course, does not mean that mind-stuff do not also differ. However, it is their coming together in real time and space that matters so far as material engagement is concerned. This ontological gathering, the coming together of specific mind-stuff I call *thinging* (Malafouris 2014, 2016a).

The specific argument that MET is putting forward in this connection is that, more often than not, our ways of think-ing are better described as modes of *thing-ing*. To explain: thinking is usually understood as something we do *about* things in the absence of things. On the contrary *thinging* denotes the kind of thinking we do primarily *with* and *through* things. For the material engagement approach *withness* and *throughness* takes precedence over *aboutness.*

I should clarify that in the context of MET the term ‘things’ is used in the broad sense of material forms, socio-material assemblages and techniques – it refers to the materiality of objects and artefacts as much as it refers to the materiality of space and the built environment. I have coined the term *thinging* to articulate and draw attention into the specific varieties of cognitive life instantiated in “actual occasions” of thinking and feeling *with, through,* and *about* things. The notion of *thinging* signifies the mentioned Peircean ontological *synechism* (continuity – from the Greek *synechēs*, meaning continuous) between mind and matter. In other words, I use the term things to signify energetic compounds of form and matter, and the term *thinging* to signify flow: the ongoing movement and transformation of mind-stuff. *Thinging* should not be understood as a psychological process of internalization or representation by which things become the *object of consciousness.* Rather *thinging* should be seen itself as an *act of consciousness.* The philosopher Martin Heidegger, in his famous essay “Das Ding” (The Thing) (1975, 166), uses the same term, i.e., “thinging” to express how things “gather” space and time, tying together their material constituents. The meaning of the term *thinging* in the context of MET retains this original sense of ‘gathering’ but it also diverge from the Heideggerian phenomenological path in many respects. More than a re-construal of the Heidegerian being-in-the-world, the process of material engagement essentially refers to a process of *becoming with* and *through* the world, leaning towards Alfred North Whitehead’s notion of *prehension* (1978; Litman 1947) and John Dewey’s ‘transactional’ sense of ‘situation’ (Dewey 1989[1929]; see also Gallagher 2009).

Important to note is that with *thinging* the focus falls on process ontologies and ecologies (Ingold 2012; Hutchins 2010b; Bateson 1972) rather than on static decontextualized objects, tools or other material structures. The *hylonoetic* field of pottery making I will discuss in the next section will be used to exemplify that. As we will see, within this *hylonoetic* field it makes no sense to raise questions about isolated entities and passive representations. It hardly makes any sense asking, for instance, what is it to be a potter, a vase, or clay. It only make sense to think of process and flow, asking questions about what the clay and the human hand can do acting together, in partnership, or about how exactly they relate and connect to each other, or about what they affect, about what they become, about what they bring forth. In doing so *thinging,* on the one hand, frees thinking from a cognitivist view of what mind consists of, and on the other, frees things from a narrowly modernist definition of what matter consists of. The analytical value of the notion *thinging*, then, lies in helping us to understand not what things are (as entities), but instead, how things come to be (as ‘events’), that is, how things come to possess ontological specificity or multiplicity in the course of their life history. Thinking is not the cause of our *thinging*. Rather, the two are inseparable: thinking is *thinging*. There are no two separate processes, one realised on the ‘inside’ and the other on the ‘outside’, but a single process of cognitive becoming. This description of *thinging* opens up new possibilities for exploring what minds and things are, and how they relate and connect to each other.

Things have a cognitive life not because of what they represent, or of how they can be represented, but for what they *do*. Things are present in the process of mind. For instance, things play an important part in the integration and coordination of processes that operate on radically different time scales (e.g., neural, bodily, cultural, and evolutionary). Through their physical persistence, they help us to move across the scales of time and to construct bridges between temporal phenomena that operate at different experiential levels. They also work best over the long term, accumulating biographies through joint participation in cultural practices, in ways that often escape the temporal limits and rhythms of individual experience.

Things become the non-biological stuff of mind on a par with other biological stuff like bodies and neurons. However, the parity I am talking about here, although related, is not the same with the parity found at the heart of extended mind theorists. In the case of MET, the starting point is not the mind as we know it from the ‘inside’ but a relational or extensive mind as we know it from ‘outside’. Thinking *about* presupposes the thinking *with* and *through* (both evolutionary and developmentally).^7^ This I call primacy of material engagement. We humans are *thingers*.

To account for that MET embodies a distinctive anthropological interest in the comparative study of materiality, materials and techniques (Ingold 2012, 2013; Knappett 2005; Knappett and Malafouris 2008; Latour 1990, 1992, 1999; Suchman 2006) which it blends with perspectives from postphenomenology (Idhe and Malafouris 2018; Verbeek 2005). Moreover, MET shares with New Materialism(s) a special ‘attentiveness’ to things, as well as an interest in understanding the ‘vitality’ and the ‘mattering’ of mater, topics that have been developed especially through the work of Barad’s agential realism and Bennett’s thing power (Barad 2007; Bennett 2010). However, with MET, the primary focus remains on the process ontology of human becoming seen as the creative generation of hylonoetic events and material signs. To understand the entangled spirit of matter we need to return to the basics: fire, air, earth, and water. If that sounds far-fetched, or too complicated, let me put it more simply using a specific example of such an entanglement in the form of clay.

## From thinking to thinging in pottery making

We started this article by drawing a line. We have used this minimal creative gesture as a means to rethink the boundaries of the mind from a material engagement perspective. We now turn to a different domain of creative action, especially fruitful for observing *thinging*: ceramics and the craft of pottery making. The morphogenetic plasticity of clay offers a paradigm case, not just of line-making in three dimensional form, but also of the *hylonoetic* field of thinking through and with things that we are trying to understand. Close, participant observation of the potter’s hand allows us to attend the transactional logic of active touch and creative gesture. This will help us to make some progress with our questions about how are things related to thinking and what kinds of marks are marking the mental? For specific examples I draw on my comparative anthropological and ethnographic study of pottery making in Greece (Malafouris 2008c, 2013, 2014; Malafouris and Koukouti 2017, 2018; but see also March 2017).

**Fig. 1:**
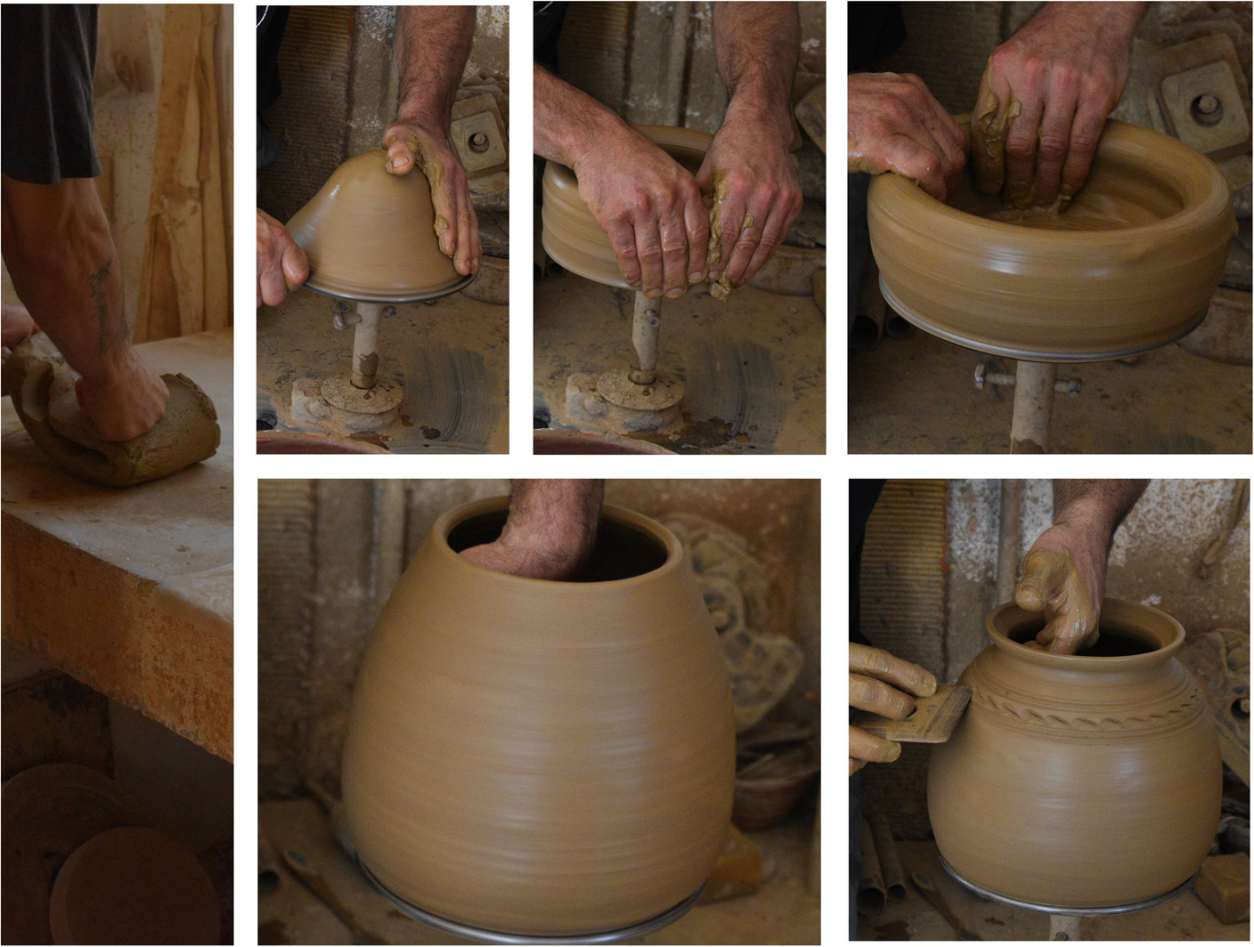
Thinking *with* and* through* clay

Look at the image of the ceramic vase that is being formed by the hands of the potter on the wheel (Fig. 1). Try to visualise, to the extent that the image and previous experience allows, the flow in-between the hand of the potter and the affordances of clay on their way to produce form. The image depicts what Whitehead (1978) would call a processual “event” in the making and life history of this material form. The plastic form on its way to the kiln exists somewhere *in-between* the states of matter and form. It preserves enough stability to be identified as a specific form. Still, it retains its plastic nature which means that is open to further change and transformation.

Where does the thinking of the potter end and the forming of clay begin? What is the boundary that supposedly separates pure mind-stuff from the non-mental physical stuff? Different, perhaps more general, versions of those questions have been raised in different fields.^8^ Still, trying to answer them from the perspective of a participant observer in the case of pottery making, they become meaningless. The reason for this is simple: these questions although deeply sedimented in the orthodoxy of philosophy and cognitive science are nonetheless resting on certain implicit assumptions about the nature and boundaries of human intelligence that are fundamentally untenable at the level of mediated action. As we discussed at the beginning of this article, traditionally, the received view of the human mind has been that of an internal brain-bound device operating primarily by constructing and manipulating internal representations of the outside world. This so-called ‘cognitivist’ view of mind has been challenged and is changing (see for review Malafouris 2013; Gallagher 2017) but remains the norm still. Yet, even if such an assumption persists, it is certainly falsified by what we observe when we actually study pottery making, where clearly throwing and thinking are inseparable (see for more detailed treatment Malafouris 2008c, 2013, 2014). It is simply wrong to assume that the potter’s head, or the brain that lies therein, offers a ‘natural’ demarcation line for separating pure mind-stuff from the clay and all the other inanimate material-stuff that surround his body. Instead, what we see is a process that is profoundly embodied, situated and assembled from a variety of non-localizable mental resources and skills, spanning the boundaries of the individual brain and body.

Let’s take a closer look at the process to see what is really at stake here. Consider the following questions: What makes possible the growth of material form out of clay? What kind of bodily processes and mental recourses are involved? The usual answer would be something along the following lines: We start with the potter’s intention to produce a specific form. Then the intention is translated into a sequence of relevant motor commands. The motor commands will in turn generate a sequence of proprioceptive, tactile and visual events. Eventually, the form of the vase will emerge as a consequence of those events. Notice in this traditional representational account not only the directionality of the process from mind to matter and the vagueness of the overall description but also the total disregard of the material properties and affordances of the media involved. But what if one is to take seriously the materiality of mind-stuff? What if we try to expose their varieties, their changing relations over time, as well as the effects that that they have on the temporal structure of the potter’s experience?

Let’s look more carefully at Fig. 1. One simple way to describe this image is as if it is depicting a series of sequential events which can be seen, and talked about, as isolated occurrences, each happening one after the other, occupying a moment in time. But in reality, what may seem in the image as a sequence of linear static events is rather a dynamic non-linear continuous process. Importantly, inside this process, each of the depicted events far from being static or isolated is extending in time encompassing *retentions* from previous states in the past and *pretentions* from the future. There is temporal as well spatial continuity that unites those events. However, phenomenologically speaking, those processual events are not identical: one cannot represent, or substitute the other, although they all may stand, in some respect, for the creative process as a whole. Moreover, those single events, although continuous, do not add up to a single linear process; rather, they are non-linearly related. Relatedness is prior and dominant over ‘beingness’. This is also where Whitehead’s view of physical reality as a dynamic agglomeration of ‘events’ or ‘happenings’ irreducible to spatial extension alone meets Dynamical Systems Theory (DST) with its emphasis on the constitutive character of non-linear relations (Beer, 1995).

Even within the narrow temporal limits of this snapshot every component of the process (biological or non biological) is continuously influencing the other’s action potential. Nothing of what we see and observe here, either from the perspective of a participant observer (the anthropologist) or the observed participant (the potter) supports, or can be accounted for, by means of the usual analytical divides of subject/object, mind/matter, nature/culture etc. Instead, what we see and experience is a mode of cognitive becoming. That is, we see a flow of energies within and between varieties of materials. This is how energies are being transformed into agencies. Agencies when embodied in living bodies can also acquire experiential content and sometimes develop awareness, i.e., a ‘sense’ of agency. But this awareness of agency, characteristic of human bodies, is largely an illusion. There is no agent apart from the action. The clay moves with the hands and the hands move with the clay. Agency is not a permanent feature or property that someone (human or non human) has independently of situated action but the emergent product of material engagement seen in our image as a creative tension of form and flow. What we call an agent refers to a momentary gathering or anchoring of several perspectival qualities that varies with time and cannot be fixed state *a priori* (see also Malafouris 2008c). The potter’s skill lies precisely in discovering the right balance of agency for each specific stage in the process of form-making. The creation of a new form on the wheel brings about a re-working of the potter’s imagination and ways of seeing as well as a new understanding of the agentive capacities and vitality of clay.

As a consequence of that it can be argued that the form we see emerging in the picture is not the result of human intentionality; rather, material form is folded into the mental by means of *prehension.*^9^ That is to say, the form of the vase enfolds in its constitution the totality of the forces, energies, memories, skills and relations of which it is the momentary outcome. It is as if the potter’s intentions inhabit the clay and the affordances of clay bring forth the potter’s intentions.

Our example of pottery making helps us to rethink the creative tension of form and flow in a way that escapes the old ontological splitting between matter and mind. The mind is not imposed or opposed to matter. Rather, mind and matter merge together in the activities and experience of the situated bodies that carry forward the process of thinking. What we see is not mind or matter; nature or culture; it is instead a way of thinking and feeling with and through the soft clay.

One may protest that the handling of clay, even in sculptural ceramics, is not sufficiently ‘representational’ to count as a paradigmatic example of human thought processes such as memory, imagination and creativity. However, I argue that the throwing of a pot on the wheel gathers, in a single continuous act of material transformation, all the basic processes that phenomenology and cognitive science take as their object of study.

There is no deficiency of higher intelligence in pottery making. Quite the contrary, pottery making, like the rest of human arts and crafts, bring forth, enact and re-create precisely the form of intelligence that drives human cognitive evolution. It is not the movement of clay that is lacking creative consciousness, memory or imagination. It is we, as modern observers that often lack the ability or the appropriate methodologies to follow that movement and to understand the cognitive life it entails. If we cannot see the mind in clay, it is because of our deeply entrenched assumptions about the location and ontology of mind stuff. My claim is not that the potter is exercising ‘higher’ cognitive abilities in order to produce a good pot. Neither am I making a claim about embodiment or cognitive capacity. My claim is about cognitive ecology; it does not refer to what happens inside the mind or brain of the potter as he or she forms the clay; rather it refers to what happens *in-between*. It calls attention to the ways by which acts of throwing bring about unique form of handmade intelligence and imagination.

## Conclusions

Phenomenology and the cognitive sciences have long reached an agreement that mental events do not occur in a vacuum or some a priori metaphysical space. They are better described as components of our lived experience, the skills and capacities of our bodies. New radically embodied and enactive frameworks are pushing this idea even further changing the way we think about the mind (Hutto and Myin 2013; Di Paolo et al. 2017; Gallagher 2017; Newen et al. 2018). Still, radical or not, those frameworks remain largely undecided about how exactly to delimit those living occurrences of mentality from their surrounding material environment and how best to understand their material bases (see Malafouris 2018). For MET, the question ‘what things are?’ and the question of ‘what minds are?’ are inseparable. The main contribution of MET is precisely to change the way we think about the relation between cognition, affect and materiality, or else the co-constitution of people and things. We are used to think about things as inert and passive. MET see things as dynamic, perturbatory, mediational means whose presence has the potential of altering the relationships between humans and their environments. New artefacts create novel relations and understandings of the world. New materialities bring about new modes of acting and thinking.

In that sense, MET diverts from the classical phenomenological programme that prioritise subjective experience over situated action. The human capacities of agency, memory and imagination are seen as distributed material processes extending beyond the individual. Those capacities are no longer seen to exist only in the interiority of the human brain. An ecology of mind thus emerges: one in which notions of material agency, material imagination, or material memory gain new meaning and ontological significance. Perhaps, the term ‘material’ may seem unnecessary. What would be the meaning of ‘immateriality’ in this context? Yet, materiality matters because it refers to more than mere matter. It refers to the constitutive intertwining of mind with matter.

As we discussed, and as many of the articles in this special issue illustrate, a distinctive feature of MET lies in its conviction that minds and things are continuous and inter-definable processes rather than isolated and independent entities. Such a position has serious ontological and epistemological consequences that demand a thorough rethink of the actual ways we practice cognitive science and make sense of human intelligence. The drawing of a line, the making of a stone tool, or the forming of a clay vase provide a unique perspective for understanding the enactive and material bases of human thought as it becomes constituted in deep time history. Material Engagement Theory offers the means to interrogate those elementary practices and their transformations in time.

## Footnotes

1 For a comparative anthropology of the changing relations between lines and surfaces and their significance in human life see the work by Tim Ingold. Ingold (2016). *Lines: a brief history*. Routledge.

2 Lines, of course, can also become, depict, or represent all kind of things depending on context and semiotic intent. But for my purposes in this example let’s assume that they don’t. I am after something more basic here.

3 For some good reviews of the recent trends and developments in this rapidly developing cross-disciplinary field see Renfrew et al. 1994; Malafouris 2009; Malafouris and Renfrew 2008, 2010; Renfrew 2004; Renfrew et al. 2008; Iliopoulos and Malafouris 2014; Knappett 2005; Coolidge and Wynn 2018; Wynn and Coolidge 2017.

4 Especially his thesis of connectedness that sees matter and energy imbued in dynamic informational patterns of difference and connectivity.

5 Comes from the Greek words (*hyle*) for matter and (*nous*) for mind. See discussion in Malafouris (2016c). Hylonoetics: on the priority of material engagement. In K. Grigoriadis (Eds) *Mixed Matters: A Multi-Material Design Compendium* (pp. 140–146). Jovis Verlag.

6 In recent years Material Engagement Theory has been put to the test in various contexts ranging from the study of enactive intentionality in early lithic technologies (Malafouris 2012a, b; Overmann and Wynn 2018; Garofoli 2015; Chakrabarty 2018) and the evolution of material signification and symbolism (Renfrew 2001; Iliopoulos 2016, 2017; Malafouris 2008a, b), to the debate over human origins and the meaning of behaviourally modernity (Garofoli 2016a, b; Iliopoulos and Garofoli 2016; Garofoli and Iliopoulos 2017; Roberts 2016); and from the development of numerical cognition (Overmann 2016a, b, c, 2017, 2018; Malafouris 2010c) and the development of early writing systems (Overmann 2017; Malafouris 2012b) to the study of creativity (Malafouris et al. 2014; Walls and Malafouris 2016); also from  the study of memory and material imagination (Malafouris 2015; Malafouris and Koukouti 2018); embodied intersubjective interactions and joint action (Gallagher 2018; Gallagher and Ransom 2016); craft and art (Arnold 2018; Malafouris and Koukouti 2017; March 2017) to the study of digital design (Poulsgaard and Malafouris 2017; Poulsgaard 2017), media (Clowes 2018; Aydin et al. 2018) and mental health (Yatczak 2018).

7 We are thinking with and through ‘representations’ in the form of material signs long before we made them our yardstick for what counts as ‘cognitive’. Of course, we can imagine and represent things but that is not a separate process but the culmination of the previous two.

8 Famously by the anthropologist Gregory Bateson (1973) and by the philosophers Andy Clark and David Chalmers in their famous essay The Extended Mind (1998).

9 Here I am referring to Whitehead’s sense of ‘prehension’ which can be described as a kind of creative border thinking that blends time (past, present and future), feeling and memory (see also Gosden and Malafouris 2015, 4–6). This prehensive temporal binding, and the material consciousness that comes with it, is immanent not just in the potter’s brain/body but in the ongoing feedback loops between biological and non-biological materials, energies and surroundings.
